# Use of Optical Coherence Tomography and Optical Coherence Tomography Angiography in the Diagnosis and Follow-Up of Endogenous *Candida* Endophthalmitis: A Case Report

**DOI:** 10.3390/medicina60020207

**Published:** 2024-01-25

**Authors:** Agnieszka Kubicka-Trząska, Dawid Bugara, Katarzyna Żuber-Łaskawiec, Weronika Pociej-Marciak, Anna Markiewicz, Bożena Romanowska-Dixon, Izabella Karska-Basta

**Affiliations:** 1 Department of Ophthalmology, Faculty of Medicine, Medical College, Jagiellonian University, ul. Kopernika 38, 31-501 Krakow, Poland; dawid.bugara@su.krakow.pl (D.B.); katarzyna.zuber-laskawiec@uj.edu.pl (K.Ż.-Ł.); weronika.pociej-marciak@uj.edu.pl (W.P.-M.); anna.markiewicz@uj.edu.pl (A.M.); bozena.romanowska-dixon@uj.edu.pl (B.R.-D.); izabella.karska-basta@uj.edu.pl (I.K.-B.); 2 Clinic of Ophthalmology and Ocular Oncology, University Hospital, ul. Kopernika 38, 31-501 Krakow, Poland

**Keywords:** optical coherence tomography, optical coherence tomography angiography, endogenous *Candida* endophthalmitis, macular neovascularization, intravitreal injection

## Abstract

*Background:* Endogenous *Candida* endophthalmitis (ECE) is a rare but sight-threatening disease. Patients with ECE present with various clinical signs and symptoms, which can complicate the diagnosis. The aim of this report was to demonstrate the outcomes of treatment and to diagnose macular complications caused by intraocular inflammation. *Case presentation:* A 41-year-old woman with a history of acute intermittent porphyria presented with a progressive vision loss in her left eye. Left-eye OCT revealed findings consistent with a fungal etiology, which was confirmed by the culture of swabs collected from a central vein catheter. The outcomes of intravenous fluconazole treatment were not satisfactory, and the patient developed recurrent attacks of porphyria, suggesting a porphyrogenic effect of systemic antifungal therapy. Repeated intravitreal injections with amphotericin B led to a gradual regression of inflammatory lesions. However, follow-up examinations revealed active macular neovascularization (MNV) on both OCT and OCTA scans. The patient was administered intravitreal bevacizumab. At the 11th month of follow-up, OCT and OCTA scans showed significant inflammatory lesions regression with macula scarring, and no MNV activity was detected. *Conclusions:* This case highlights the importance of OCT and OCTA as valuable noninvasive imaging techniques for the identification of ECE, the monitoring of its clinical course, and the diagnosis of macular complications.

## 1. Introduction

Endophthalmitis is a purulent inflammation of the inner layers of the eye with exudation within intraocular fluids (vitreous and aqueous humor) resulting from intraocular colonization by microorganisms. It is a rare but potentially vision-threatening disease. Based on the mode of entry of microorganisms, it is divided into exogenous and endogenous types [1,2]. The vast majority of endophthalmitis cases have an exogenous origin. The infection may occur after intraocular surgical procedures (cataract surgery, trabeculectomy, intravitreal injections, keratoplasty), penetrating ocular trauma, or pathogen infiltration following infections of the cornea (keratitis, corneal ulcer) [3,4,5,6,7,8]. On the other hand, endogenous endophthalmitis is caused by the hematogenous dissemination of microorganisms from the primary site of inflammation [2,9,10]. It represents only 2% to 8% of all endophthalmitis cases [2]. Several risk factors for endogenous endophthalmitis have been identified, including indwelling catheters, prolonged hospitalization, diabetes, immunosuppression, renal pathology, human immunodeficiency virus infection, influenza A virus infection, intravenous drug abuse, or malignancies [2,9,10,11,12,13]. The primary pathological factor may be either bacterial or fungal. In fungal endophthalmitis, *Candida albicans* stands out as the most prevalent etiological factor, followed by *Cryptococcus neoformans*, *Aspergillus* spp., and *Paecilomyce* spp. [14] Recent reports have indicated that the incidence of endogenous fungal inflammation in patients with fungemia has decreased significantly and does not exceed 2.2% [15]. However, its prevalence depends on a geographical region. A meta-analysis conducted by Phongkhun et al. [16] showed that the prevalence of fungal endophthalmitis reported in studies from Asian countries was two- and four-fold higher as compared with studies from European countries and as reported by the American Academy of Ophthalmology (AAO).

Endogenous *Candida* endophthalmitis (ECE) is a serious sight-threatening disease. Bilateral involvement is observed in more than 25% of cases [10,15]. The diagnosis of ECE is difficult and is based on clinical findings supported by additional diagnostic tests, including blood culture, aqueous or vitreous culture, and microscopic examination of the sample. However, the sensitivity of these tests is limited [17,18,19,20]. Techniques based on the amplification and quantification of DNA, such as a polymerase chain reaction test, were shown to be more sensitive than culture methods, but a low microorganism load in a sample can yield false negative results [21].

Along with advances in intravitreal medical treatment, there has also been an ongoing improvement in modern imaging techniques such as optical coherence tomography (OCT) and OCT angiography (OCTA). These tools allow for a noninvasive imaging of the retina and choroid, enabling an early diagnosis and immediate treatment of numerous diseases, especially those involving the macula. Recently, it was also shown that the characteristic findings obtained via OCT facilitate a correct diagnosis in ECE cases [22,23,24,25].

This case report highlights the usefulness of OCT and OCTA in the diagnosis and follow-up of ECE in a patient with a history of prolonged hospitalization for severe intermittent porphyria, leading to the placement of a permanent central vein catheter 3 weeks before the onset of visual symptoms.

## 2. Case Description

A 41-year-old woman was admitted to the Metabolic Disorders Unit of the University Hospital in Kraków, Poland, due to a life-threatening attack of acute intermittent porphyria with severe visceral manifestations. The patient was treated with an intravenous injection of hemin and morphine. Due to peripheral venous access problems, an internal jugular vein cannulation was performed, and the treatment was continued. During systemic therapy, the patient developed a fever and started to complain of blurred vision in the left eye. Systemic antibiotic therapy with meropenem at a dose of 2 g every 8 h was administered. However, the patient complained of a further deterioration of vision, pain, and photophobia in her left eye.

**Figure 1:**
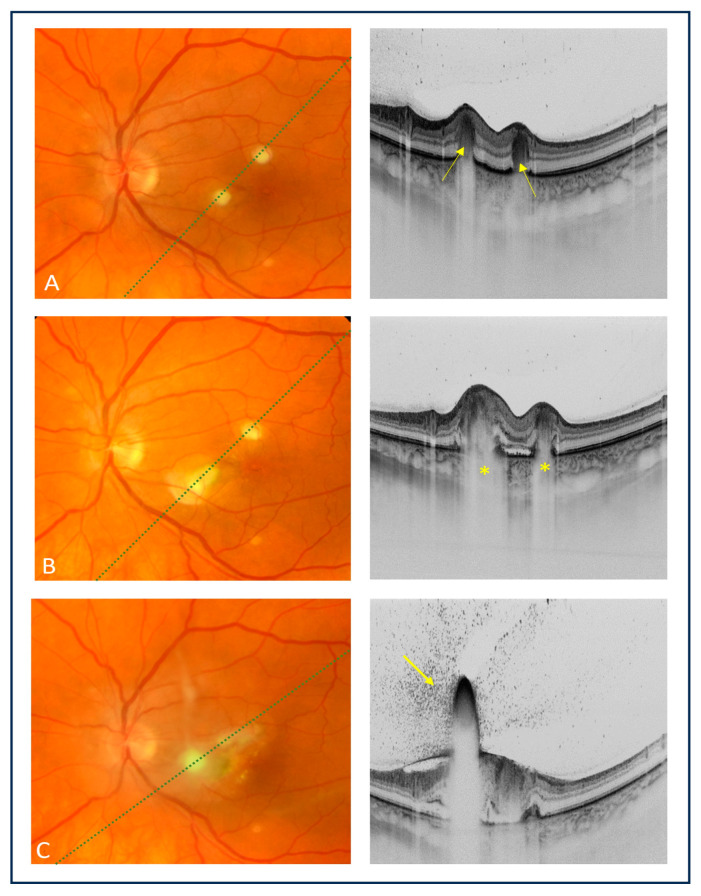
Color fundus images of the left eye (left column) and corresponding OCT cross-sections (right column). (**A**) Baseline examination: fundoscopy showed two small creamy-white inflammatory foci involving the macula; OCT showed two hyperreflective lesions involving mainly the outer layers of the retina (yellow arrows); (**B**) fundus photograph showed the worsening of the inflammatory lesions at 5 days; OCT showed hyperreflective infiltrates involving the entire retinal thickness with deeper layer shadowing (“rain-cloud” sign) (yellow asterisks); (**C**) fundus photograph showed the progression of inflammatory foci at 6 days; OCT showed the spread of one lesion over the retinal surface, a characteristic hyperreflective preretinal aggregate obscuring the underlying retina and inflammatory punctate vitreous opacities extending from the lesions to the posterior hyaloid, referred to as the “inverted snowing-cloud” sign (yellow arrow).

The patient was consulted at the Department of Ophthalmology and Ocular Oncology of the University Hospital in Kraków. On ophthalmic examination, the best corrected visual acuity (BCVA) was 1.0 in the right eye and 0.8 in the left eye. No abnormalities were present on a slit-lamp examination of both eyes. The fundus examination of the right eye showed no pathology. However, in the macula of the left eye, there were two creamy-white, consolidated inflammatory lesions. Multimodal imaging using fundus photography (Topcon TRC 50DX fundus camera, Topcon Corporation, Tokyo, Japan) and swept-source OCT (Topcon DRI OCT Triton, Topcon Corporation, Tokyo, Japan) revealed hyperreflective lesions involving the choroid and outer retinal layers, corresponding to the inflammatory foci found via fundoscopy (Figure 1A). During the follow-up, the patient experienced a recurrent attack of acute intermittent porphyria, suggesting that meropenem could be a porphyrinogen agent that triggered the attack. The ophthalmic examination performed 5 days later showed further deterioration of vision and the progression of inflammatory chorioretinal inflammatory foci via both fundoscopic and OCT examinations (Figure 1B).

**Figure 2:**
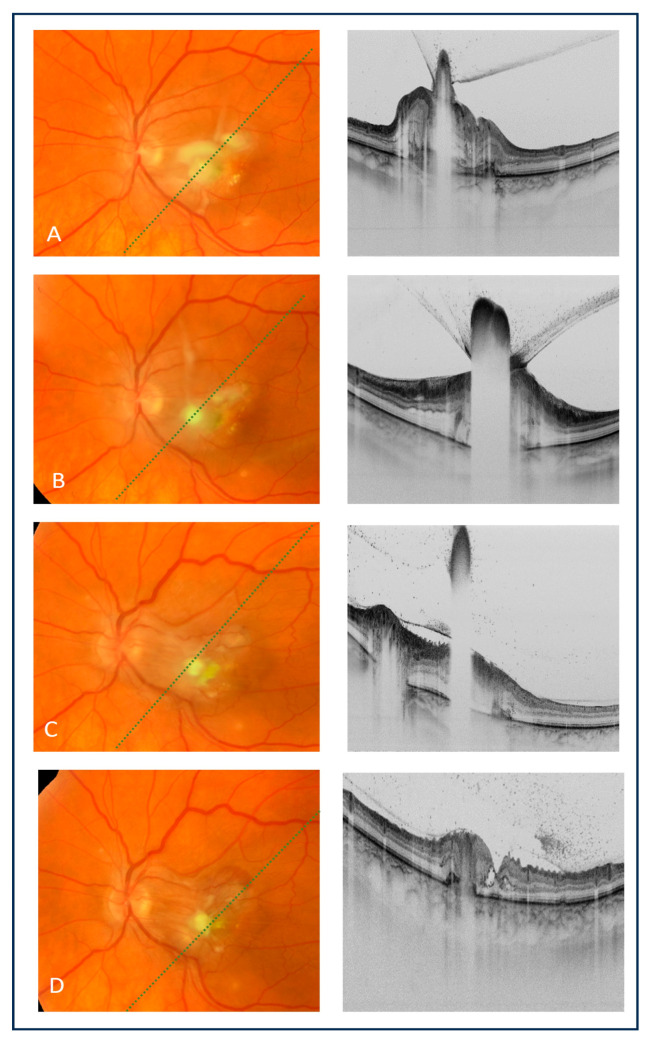
Color fundus images of the left eye (left column) and corresponding OCT cross-sections (right column). (**A**) Fundoscopy and OCT scans performed on the day of the first intravitreal injection of amphotericin B showed worsening of the chorioretinal inflammation as compared with the examination 3 days earlier (see Figure 1C); (**B**) seven days after the intravitreal injection, a significant regression of inflammatory lesions was observed, and OCT demonstrated consolidation of the inflammatory foci. Owing to good response, the second intravitreal injection of amphotericin B was administered; (**C**) seven days later, further regression of chorioretinal inflammatory foci with posterior vitreous detachment was noted on OCT, and the third injection of amphotericin B was administered; (**D**) after another seven days, the inflammatory foci further regressed, but the development of an epiretinal membrane was noted. The patient received the fourth injection of amphotericin B.

Considering the clinical presentation and imaging findings, a fungal etiology of intraocular inflammation in the left eye was suspected. The blood culture from the internal jugular venous catheter was positive for *Candida albicans*, thus confirming the diagnosis of endogenous fungal endophthalmitis. Intravenous therapy with fluconazole at a dose of 400 mg/day was initiated. However, due to an underlying disease (porphyria), fluconazole was poorly tolerated. The patient experienced another acute attack of porphyria. The results of systemic therapy also proved to be unsatisfactory, as the chorioretinal lesions continued to deteriorate clinically (Figure 1C). The follow-up showed progression of inflammatory lesions compared to the ophthalmic examination performed a week earlier (Figure 2A). Moreover, vitreous exudation over the posterior pole was noted, and the BCVA of the left eye decreased to 0.16. Based on the antibiotic susceptibility using antibiogram results and in accordance with the local Bioethics Committee approval, the patient was referred for intravitreal injections of amphotericin B (5 μg/0.1 mL). The use of intravitreal voriconazole, which is also recommended for ECE, had not been considered and approved by the local Bioethics Committee. A total of four amphotericin B injections every 7 days were administered.

Intravitreal amphotericin B administration resulted in the regression of inflammatory foci and vitreous exudates, as revealed by an fundoscopy and OCT. However, the BCVA of the left eye remained unchanged. The chorioretinitis resulted in the loss of the retinal pigment epithelium, photoreceptors and outer retinal layers with foveal scarring involving the whole retinal thickness, and the post-inflammatory epiretinal membrane was also observed (Figure 2A–D).

**Figure 3:**
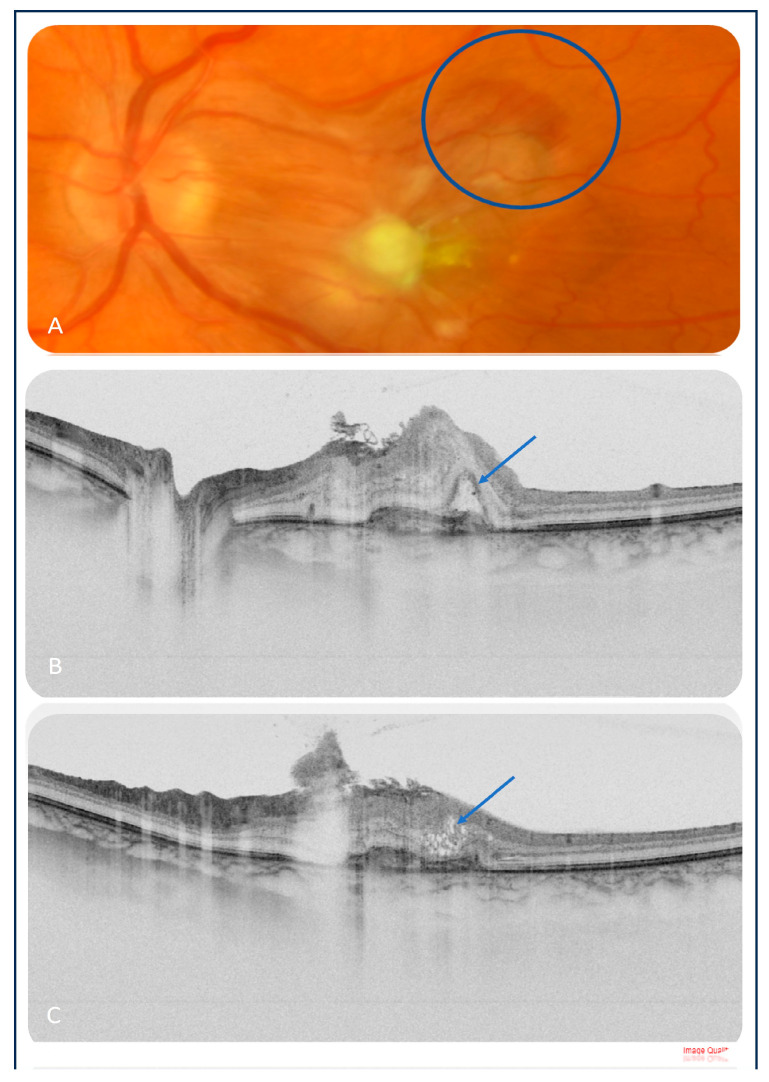
(**A**) Color image of the left eye 20 days after the fourth intravitreal injection of amphotericin B showed a significant regression of the chorioretinal lesions with epiretinal fibrosis and retinal hemorrhage in the upper-temporal aspect of the macula (blue circle); a suspicion of iMNV was raised. (**B**,**C**) OCT scans revealed active iMNV, and subretinal and intraretinal fluid can be seen (blue arrows).

**Figure 4:**
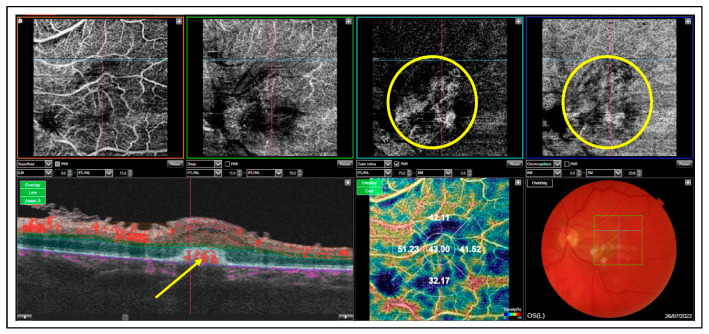
OCTA scans revealed vascular network corresponding to iMNV (yellow circles). Corresponding B scan showed subretinal lesion with flow detection (yellow arrow).

Three weeks after the fourth intravitreal injection of amphotericin B, a small hemorrhage in the upper-temporal part of the macula was noted (Figure 3A), and the patient complained of more pronounced metamorphopsia and more blurred central vision in her left eye (the BCVA was counting fingers). An OCT scan showed the presence of intraretinal cysts, subretinal fluid and a hyperreflective subretinal lesion suspected of inflammatory macular neovascularization (iMNV) (Figure 3B,C). As there were difficulties with the vascular access, fluorescein angiography could not be performed. Moreover, the patient was in a poor general condition. Therefore, OCTA (Topcon OCTA Triton, Topcon Corporation, Tokyo, Japan) was conducted, which confirmed the diagnosis of active iMNV (Figure 4).

**Figure 5:**
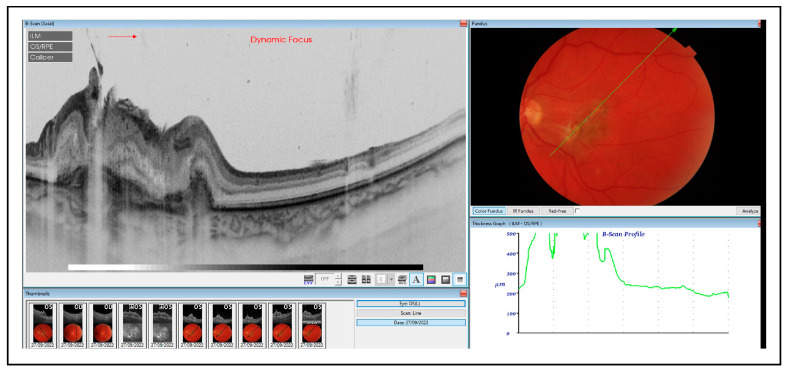
OCT image revealed inactive iMNV with regression of intraretinal and subretinal fluid. The post-inflammatory choioretinal scar was seen.

The patient was scheduled for an intravitreal injection of bevacizumab (1.25 mg/0.05 mL) in the left eye. The OCT examination performed 4 weeks after the anti-VEGF intravitreal injection revealed anatomical improvement. The regression of intra- and subretinal fluid was observed, and visual acuity of the left eye remained poor, as assessed based on counting fingers (Figure 5). The total follow-up period was 11 months.

## 3. Discussion

The diagnosis of ECE may be challenging. Previous studies have demonstrated that endogenous endophthalmitis can be missed in 16% to 63% of cases [9,26]. Therefore, it is necessary to raise the awareness about this condition among clinicians, especially in the presence of risk factors for ECE.

As the choroid is a rich vascularized tissue, it is the primary site affected by fungal infection. Subsequently, the inflammation spreads and progresses anteriorly from the choroid to the outer and then to the inner retinal layers. Consequently, endogenous fungal endophthalmitis often presents with choroiditis or chorioretinitis. As the infection worsens, it involves the vitreous body [26,27]. Progression is typically slow. *Candida* chorioretinitis typically manifests as creamy-white, well-circumscribed chorioretinal lesions accompanied by white vitreous opacities, representing the characteristic “string of pearls” sign [14,27]. There are only single reports describing endogenous *Candida* chorioretinitis progression to endophthalmitis [28].

The differential diagnosis includes noninfectious uveitis (e.g., in association with sarcoidosis, Behçet syndrome, sympathetic ophthalmia, or Vogt–Koyanagi–Harada disease) as well as infectious entities such as bacterial endophthalmitis, tuberculosis, syphilis, herpes virus infections, toxoplasmosis, and masquerade syndromes (intraocular lymphoma) [14,15,29].

A diagnostic vitrectomy with laboratory workup of vitreous specimens is a useful tool in establishing the definitive diagnosis in uveitis with atypical phenotypes and atypical clinical course [30]. In our patient, the diagnosis of *Candida* chorioretinitis was based on typical clinical and imaging findings, consistent with prior reports, and on a positive blood culture test for *Candida albicans* from the internal jugular venous catheter [14,22,23,24,25,26,27,28].

Recently, it has been shown that characteristic OCT findings may facilitate the correct diagnosis of fungal infections in cases with ECE [22,23,25]. Invernizzi et al. [22] described two distinct patterns of choroidal and retinal involvement: intraretinal and chorioretinal, influencing the final BCVA, which was shown to be worse in eyes with the chorioretinal lesion. Additionally, all eyes with ECE presented with hyperreflective preretinal lesions that obscured the underlying retina and created a shadowing effect called the “rain-cloud” sign. Chorioretinal involvement was noted in our patient, along with the “rain-cloud” sign on OCT images as a peculiar feature of fungal inflammatory foci.

Anvari et al. [25] used the term “inverted snowing-cloud sign” to describe a preretinal lesion with vitreous aggregates resembling a white cloud with snowflakes. This finding was also noted in a described case, corresponding to the more advanced stage of fungal inflammation extending to the vitreous body. However, according to the authors, the “inverted snowing-cloud” sign may not be specific for *Candida* endophthalmitis and can be observed in other retinal and choroidal diseases associated with vitritis.

Zhuang et al. [23] described the following four types of OCT manifestations of ECE: type 1 representing subretinal lesions in the macula, type 2 lesions located in the inner retinal layers, type 3 lesions involving the full-thickness retina, and type 4 representing the subinner limiting membrane lesions. After antifungal treatment, the final visual acuity of eyes with type 2 lesions was improved [23]. Based on this classification, in our patient, we observed type 3 lesions, which translated into a poor prognosis for the affected eye.

The treatment strategy for patients with ECE depends on the clinical presentation and includes systemic therapy with fluconazole or voriconazole, intravitreal injections of amphotericin B or voriconazole, and–in more complicated cases–vitrectomy [31]. There is evidence that systemic therapy with fluconazole and voriconazole is effective in patients with ECE [32,33]. While it is thought to have a generally good safety profile with only a few side effects, it may have a significant impact on the clinical course of acute intermittent porphyria [34]. Antifungal agents also may have a porphyrogenic effect and trigger disease relapse [34]. Drug delivery directly at the site of the pathology is more effective than the administration of intravenous or oral medication. Moreover, local therapy is safer because it reduces the risk of systemic complications caused by the medication. This is especially important in patients who cannot tolerate systemic therapy, and exposure to the therapy may cause the exacerbation of systemic diseases. In our patient, systemic therapy with fluconazole triggered acute attacks of porphyria [35]. Because of poor tolerance of systemic fluconazole and macula involvement, the patient received intravitreal injections of amphotericin B. She responded well to antifungal intravitreal therapy, and the regression of intraocular inflammation was observed after four injections. However, follow-up OCT scans showed a new lesion suspected of iMNV, and OCTA confirmed the diagnosis. Therefore, a decision was made to administer the intraretinal injection of bevacizumab. Inflammatory MNV is a rare complication of chorioretinitis but is one of the most severe causes of visual impairment in this group of patients [36,37]. There are two pathophysiological mechanisms by which inflammation may promote the development of iMNV. The first one is associated with inflammatory-mediated damage of the Bruch’s membrane-retinal pigment epithelium complex, which disrupts the outer blood-retinal barrier and permits neovascular upgrowth from the choroid [28,36,38]. Another theory explains that inflammation directly compromises perfusion, generating a gradient of retinal–choroidal hypoxia that promotes formation of iMNV [36,37]. In our patient OCT imaging showed inflammatory destruction of the Bruch’s membrane–retinal pigment epithelium complex creating a locus minoris resistentiae for iMNV development. However, the involvement of other factors promoting iMNV formation in this case cannot be excluded.

## 4. Conclusions

Based on the results of our observations, OCT, as a noninvasive repeatable imaging technique, can be used to assess the response to treatment and monitor long-term outcomes in a course of ECE. On the other hand, OCTA aids a quick diagnosis and decision-making in therapy if a patient presents with secondary MNV, which is a rare macular complication of intraocular inflammation.

## Data Availability

Data supporting the findings of this study are available upon request from the corresponding author.
